# The Growing Threat of Gonococcal Blindness

**DOI:** 10.3390/antibiotics7030059

**Published:** 2018-07-12

**Authors:** Victoria Dolange, Colin P. Churchward, Myron Christodoulides, Lori A. S. Snyder

**Affiliations:** 1 Faculty of Medicine, University of Southampton, Southampton SO17 6YD, UK; victoria.dolange@agroparistech.fr; 2 School of Life Sciences, Pharmacy, and Chemistry, Kingston University, Kingston upon Thames KT1 2EE, UK; colin.churchward@googlemail.com

**Keywords:** gonococcal blindness, antimicrobial resistance, ophthalmia neonatorum, monocaprin, *Neisseria gonorrhoeae*, *Neisseria meningitidis*, gonococci, meningococci

## Abstract

Antibiotic-resistant gonorrhea is now a reality, as well as the consequences of untreatable infections. Gonococcal eye infections result in blindness if not properly treated; they accounted for the vast majority of infections in children in homes for the blind in the pre-antibiotic era. *Neisseria gonorrhoeae* infects the eyes of infants born to mothers with gonorrhea and can also infect the eyes of adults. Changes in sexual practices may account for the rise in adult gonococcal eye infections, although some cases seem to have occurred with no associated genital infection. As gonorrhea becomes increasingly difficult to treat, the consequences for the treatment of gonococcal blindness must be considered as well. Monocaprin was shown to be effective in rapidly killing *N. gonorrhoeae*, and is non-irritating in ocular models. Repeated passage in sub-lethal monocaprin induces neither resistance in gonococci nor genomic mutations that are suggestive of resistance. Here, we show that 1 mM monocaprin kills 100% of *N. gonorrhoeae* in 2 min, and is equally effective against *N. meningitidis*, a rare cause of ophthalmia neonatorum that is potentially lethal. Monocaprin at 1 mM also completely kills *Staphylococcus aureus* after 60 min, and 25 mM kills 80% of *Pseudomonas aeruginosa* after 360 min. Previously, 1 mM monocaprin was shown to eliminate *Chlamydia trachomatis* in 5 min. Monocaprin is, therefore, a promising active ingredient in the treatment and prophylaxis of keratitis, especially considering the growing threat of gonococcal blindness due to antimicrobial resistance.

## 1. Introduction

### 1.1. Ophthalmia Neonatorum

In addition to causing the sexually transmitted infection known as gonorrhea, *Neisseria gonorrhoeae* can infect the eye, where it is capable of causing ulceration of the cornea, perforation of the globe of the eye, and permanent blindness [1,2,3]. Up to 48% of infants born to mothers infected with *N. gonorrhoeae* develop ophthalmia neonatorum, and up to 10% of those who receive antibiotic prophylaxis still develop infection [2,3]. Infants can become infected in utero, and delivery via Caesarean section, in addition to carrying higher risks for the mother, does not necessarily eliminate the risk of ophthalmia neonatorum [4]. *N. gonorrhoeae* is the prevalent cause of ophthalmia neonatorum in many low- and middle-income countries (LMIC), including Malawi [5]. The development of this neonatal infection is compounded by the fact that 80% of women infected with *N. gonorrhoeae* show no overt symptoms, and even among the women visiting a genitourinary medicine clinic seeking treatment for a reproductive health concern, 50% are asymptomatic [6].

In rare cases, the closely related pathogen, *N. meningitides*, was reported to cause gonorrhea in adults and eye infections in infants [7,8]. Meningococcal ophthalmia neonatorum has the potential of developing into meningococcal meningitis and/or septicemia. Autoinoculation of the oropharynx via the nasolacrimal duct, or via purulent eyes to the hands, to the mouth, and then to the throat is also possible with gonococci and meningococci. Certainly, the risk of developing septicemia and/or meningitis from ophthalmia neonatorum caused by *N. meningitidis* is far greater than the risk of developing serious disseminated gonococcal infections.

### 1.2. Adult Eye Infections

Adult eye infections are also more regularly reported as the overall rates of gonorrhea increase worldwide. Gonococcal eye infections in developed countries are predominantly occurring in teenagers and young adults, where transmission is believed to be via autoinoculation to the eyes from genital site infections [9]. There are, however, case reports where the origin of gonococcal eye infections is unclear. For example, a 76-year-old man was diagnosed with *N. gonorrhoeae* keratitis after two weeks of worsening conjunctivitis that was unresponsive to chloramphenicol ointment. He had no genital co-infection, and claimed to have had no sexual activity for 40 years [10]. Even more intriguing, are the repeated outbreaks of *N. gonorrhoeae* conjunctivitis in Aboriginal communities in Australia, reported over a span of more than 60 years [11,12]. The gonococcal isolates from the eye appear to have come from a single lineage that was distinct from genital isolates in the region [13]. In Ethiopia, there were 9000 cases of gonococcal eye infection within 8 months in 1987–1988 [14]. Many of these cases were in children under the age of five, but a few were in neonates. In both Australia and Ethiopia, there was no concurrent genital outbreak, and it was suggested that transmission was via flies following peak periods of rain [11,14].

### 1.3. Treatments for Ophthalmia Neonatorum

Ophthalmia neonatorum can spread from the eyes to cause systemic infections such as gonococcal arthritis, and potentially life-threatening septicemia and meningitis [15]. For this reason, it can be beneficial to treat the eye infection with antibiotics administered systemically, as well as topically. However, antibiotic-resistant strains of *N. gonorrhoeae* are now a major threat to human health [9,16], and their emergence and spread reduce the available systemic antibiotic treatment options for all gonococcal diseases, including eye infections. Therefore, rapid elimination of colonizing gonococci immediately after birth via an effective prophylaxis may be the best solution for infant health in the face of multi-drug-resistant *N. gonorrhoeae*. By contrast, although multi-drug resistance in *N. meningitidis* is yet to reach the levels of concern associated with *N. gonorrhoeae*, correct and rapid treatment of *N. meningitidis* is still urgent, given the severity of disease [17].

The history of treatments for ophthalmia neonatorum is shown in Table 1, and ranges from simple irrigation of the eye to the use of antibiotics and non-antibiotic antimicrobials. Resistance to erythromycin, the preferred antimicrobial prophylaxis in Canada, was 23% in 2012 (publications.gc.ca/collections/collection_2014/aspc-phac/HP57-3-2012-eng.pdf) [18]; therefore, the only available topical treatment may be ineffective. *N. gonorrhoeae* cases of ophthalmia neonatorum in Cambodia were shown to be resistant to both penicillin and fluoroquinolone, but susceptible to ceftriaxone [19], which was similarly observed with gonococcal isolates from other sites of infection [20]. There were changes in the pattern of bacterial species causing neonatal eye infections in southern China over the last 15 years, with the main Gram-negative bacteria being *N. gonorrhoeae* [21]. This trend is consistent with the increase in gonorrhea in this region.

A study of the members of the American Association of Pediatric Ophthalmology and Strabismus revealed that most clinicians (85%) treated empirically [22]. It is, therefore, key that treatments are able to kill the potential causes of infant eye infections, including *N. gonorrhoeae*, *Chlamydia trachomatis*, *Pseudomonas aeruginosa*, and *Staphylococcus aureus* [23]. Multi-species infections can also complicate treatment of bacterial keratitis. It is, therefore, preferable if an ophthalmia neonatorum prophylaxis and treatment is effective against the major causes of bacterial eye infections. Most clinicians (85%) treat topically, as well as systemically [22]. However, universal ocular prophylaxis was abandoned in Denmark, Norway, Sweden, and the United Kingdom (UK), in favor of risk-factor-based screening of mothers [24]; however, this decision may be revisited in the face of untreatable gonorrhea and the rising incidence of infection.

### 1.4. Anti-Gonococcal Properties of Monocaprin

Recently, we showed that monocaprin is a promising antimicrobial agent with the potential for use against gonococcal ophthalmia neonatorum [29,30,31]. Monocaprin is the monoglyceride of the fatty acid, capric acid. The antimicrobial activity of monocaprin was previously demonstrated against the *N. gonorrhoeae* strain, NCCP11945, and against several clinical isolates, killing gonococci at a concentration of 1 mM in 2 min [30]. Repeated passage of gonococci on media containing a sub-lethal concentration of monocaprin neither induced resistance nor genomic changes indicative of resistance arising [31]. The antimicrobial activity of monocaprin was demonstrated for other species, including efficacy against *Chlamydia trachomatis* at a concentration of 1 mM in 5 min [32].

In this short communication, we demonstrate the effectiveness of monocaprin against other bacteria capable of causing eye infections in infants and adults.

## 2. Results and Discussion

**Figure 1:**
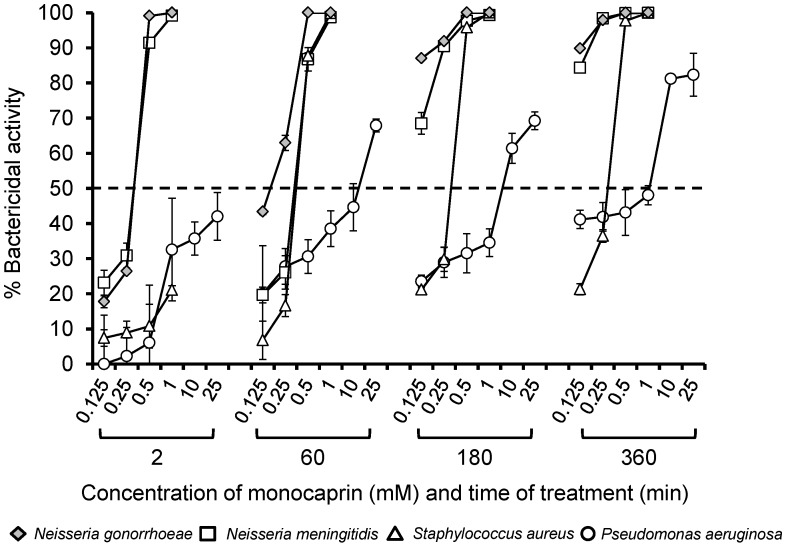
Bactericidal activity of monocaprin against a variety of different bacteria. Bacteria (~10^3^ colony-forming units (CFU) in triplicate wells) were treated with various doses of monocaprin (0.125–25 mM), and variability was determined using viable counting on selective agar at various time points (2–360 min). The symbols represent the mean bactericidal activity, calculated as the percentage of (surviving bacteria/the number of untreated control bacteria) × 100 from *n* = 2–3 independent experiments. The error bars are the standard errors of the means.

In the current study, we used a bactericidal assay to compare the efficacy of monocaprin against other bacteria that can cause ophthalmia neonatorum and keratitis [33] alongside gonococci (Figure 1). A dose of 0.5–1 mM of monocaprin killed 100% of the gonococci within 2 min with lower concentrations of 0.125–0.25 mM showing increased efficacy >60 min of treatment (Figure 1). An identical pattern of sensitivity was shown for *N. meningitidis* (Figure 1). In contrast, 1 mM monocaprin killed only 20% of *S. aureus* after 2 min, and a minimum dose of 0.5 mM monocaprin with a minimum contact time of 60 min was required to kill >80% of these pathogens (Figure 1). The highest dose of 1 mM was effective in killing 100% of *S. aureus* by 60 min (Figure 1). In contrast, doses up to 1 mM of monocaprin killed only ~50% of *P. aeruginosa* after 360 min of treatment. However, bactericidal killing of ~70% was observed after 60 min with a dose of 25 mM monocaprin, which increased marginally to 80% after 360 min of treatment (Figure 1). Previously, a different laboratory strain of *N. gonorrhoeae* produced similar results, and clinical isolates were also tested [30]; nonetheless, in the future, a broader variety of strains of these species and other multi-antibiotic-resistant species should be explored to investigate the range of susceptibilities.

The rapid activity of monocaprin against both gonococci and meningococci provides a viable topical adjunct to antibiotics for both the prophylaxis and treatment of ocular infection with these two organisms. Previous data showed that monocaprin also has rapid activity against *C. trachomatis*, lending additional support for its use against ophthalmia neonatorum. Furthermore, activity against the keratitis bacteria, *S. aureus* and *P. aeruginosa*, provides further potential clinical benefit. However, unlike these pathogens, the gonococcus is a more invasive organism, and it can penetrate intact corneal epithelia and invade underlying stroma [34].

Notably, the rapid bactericidal activity of monocaprin is unmatched by conventional antibiotics. Moreover, fatty acids such as monocaprin are believed to have multiple mechanisms of action [35], and therefore, resistance is less readily able to evolve. Various mechanisms may include the solubilization of membrane lipids, the destabilization of membrane bilayers, integration into membranes increasing permeability, the disruption of cell division, interference with nutrient uptake, and the inhibition of fatty-acid biosynthesis [35]. While the *Neisseria* species (spp.) evolved efflux pumps capable of exporting fatty acids [36,37], monocaprin is not a substrate for these pumps. Passage on media containing sub-lethal monocaprin did not select for resistance, nor did genomic changes occur that were indicative of increasing resistance [31]. In other species, resistance to the monoglyceride monolaurin was not detected in *S. aureus*, despite growth in sub-lethal concentrations for over a year [38,39], and resistance to fatty acids could not be selected for by *Helicobacter pylori* [40].

Ideally, vaccination would control gonococcal disease in general [41], but this pathogen presents considerable challenges to vaccine development, and no vaccines are currently available. There is no protective immunity, with individuals susceptible to repeated infection [42]. Viable alternatives to vaccination are needed for prophylaxis to prevent the establishment of gonococcal eye infections in regions where gonorrhea disease is prevalent. A rapid progression of gonococcal infection leads to a rapid loss of visual acuity [34]. Moreover, established infection must be rapidly eliminated to reduce the severity of vision loss and the potential need for ocular grafts [43,44]. The application of monocaprin could be a prophylaxis that halts gonococcal growth and colonization through its antimicrobial action, and therefore, such a prophylaxis would prevent the development of purulent discharge symptoms from neutrophils failing to clear the infection. Instead, the infection would be managed through the antimicrobial activity of monocaprin. There is sufficient evidence to suggest the development of monocaprin into a topical formulation that could be retained on the surface of the eye sufficiently long enough to kill the bacteria, or perhaps the potential of synergistically working with antimicrobial peptides, enzymes, and other natural defenses of the eye to clear the infection. Moreover, as an adjunct therapy, the rapid bioactivity of monocaprin could inform current treatment regimens for keratitis, and could reduce the amount of conventional antibiotics currently used to treat infections.

## 3. Materials and Methods

### 3.1. Bacteria and Growth Conditions

The growth conditions of *Neisseria gonorrhoeae* strain P9-17, *Neisseria meningitidis* MC58, *Staphylococcus aureus*, and *Pseudomonas aeruginosa* PAO1 were all described previously [45,46,47]. *Neisseria* spp. were grown on supplemented GC-agar plates [48] and *S. aureus* and *P. aeruginosa* bacteria were grown on Luria Bertani agar or nutrient agar plates. All bacteria were grown at 37 °C and 5% (*v*/*v*) CO_2_, except for *P. aeruginosa*, which did not require CO_2_.

### 3.2. Bactericidal Assay

Bactericidal activity was assessed using a slight modification of a standard method [49]. Cultures of bacteria were suspended overnight in Dulbecco’s phosphate-buffered saline, pH 7.4, and were dispensed into triplicate wells of a sterile 96-well microtiter plate, at a concentration of ~10^3^ colony-forming units per well (100 µL final volume per well). Various doses of monocaprin (Sigma-Aldrich, Saint Louis, MO, USA) were added to final concentrations of 0.125, 0.25, 0.5, and 1 mM, and the plates were incubated at 37 °C and 5% (*v*/*v*) CO_2_. Wells containing bacteria without monocaprin were included as controls. Wells were sampled at 2, 60, 180, and 360 min, and surviving bacteria were quantified using viable counting onto selective agar. The bactericidal activity of monocaprin was calculated as the percentage of (surviving bacteria/the number of untreated control bacteria) × 100.

## 4. Conclusions

Monocaprin is able to rapidly kill *N. gonorrhoeae*, *N. meningitidis*, and *C. trachomatis* [32], and has bactericidal activity against *S. aureus* and *P. aeruginosa*, making it a promising active ingredient for ocular prophylaxis and treatment to prevent the loss of vision due to eye infections. The ever-decreasing treatment options for *N. gonorrhoeae* [16], the rapid development of corneal melt and perforation [34], the potentially fatal consequences for *N. meningitidis* ocular infection dissemination [7], and the possibility of multi-species infection [22] support the development of a monocaprin-based formulation for prophylaxis and treatment.

## Figures and Tables

**Table 1:** Overview of topical treatments and prophylaxes for gonococcal ophthalmia neonatorum.

Treatment	Efficacy	Contraindications	Reference
Silver nitrate	Prophylaxis reduced cases of disease.	Chemical conjunctivitis; toxicity; failures.	[25]
Penicillin	Treatment cured disease.	Resistance developed.	[26]
1% Tetracycline	Prophylaxis and treatment cured disease. Often used in conjunction with saline washes in low- and middle-income countries (LMICs) where prophylaxis is available.	Resistance developed.	[2,5,20]
Erythromycin	Treatment cured disease.	Resistance developed.	[18]
Saline washes	May reduce accumulation of purulent discharge.	Unlikely to eliminate infection.	[5,20]
Povidone-iodine (betadine, 1.25% or 2.5% (*v*/*v*) solutions)	Prophylaxis reduced cases of disease.	5% rate of chemical conjunctivitis; failure to eradicate infection; not recommended. (https://www.cdc.gov/std/tg2015/gonorrhea.htm)	[25,27,28]
